# The temporal scale of diet and dietary proxies

**DOI:** 10.1002/ece3.2054

**Published:** 2016-03-02

**Authors:** Matt Davis, Silvia Pineda Munoz

**Affiliations:** ^1^ Department of Geology and GeophysicsYale UniversityNew HavenConnecticut06520; ^2^ Department of PaleobiologyNational Museum of Natural HistorySmithsonian InstitutionWashingtonDistrict of Columbia20004

**Keywords:** Diet, dietary proxies, isotopes, microwear, temporal scale, time averaging

## Abstract

Diets estimated from different proxies such as stable isotopes, stomach contents, and dental microwear often disagree, leading to nominally well‐supported but greatly differing estimates of diet for both extinct and extant species that complicate our understanding of ecology. We show that these perceived incongruences can be caused by proxies recording diet over vastly different timescales. Field observations reveal a diet averaged over minutes or hours, whereas dental morphology may reflect the diet of a lineage over millions of years of evolution. Failing to explicitly consider the scale of proxies and the potentially large temporal variability in diet can cause erroneous predictions in any downstream analyses such as conservation planning or paleohabitat reconstructions. We propose a cross‐scale framework for conceptualizing diet suitable for both modern ecologists and paleontologists and provide recommendations for any studies involving dietary data. Treating diet in this temporally explicit framework and matching the scale of our questions with the scale of our data will lead to a much richer and clearer understanding of ecological and evolutionary processes.

## Introduction

**Figure 1:**
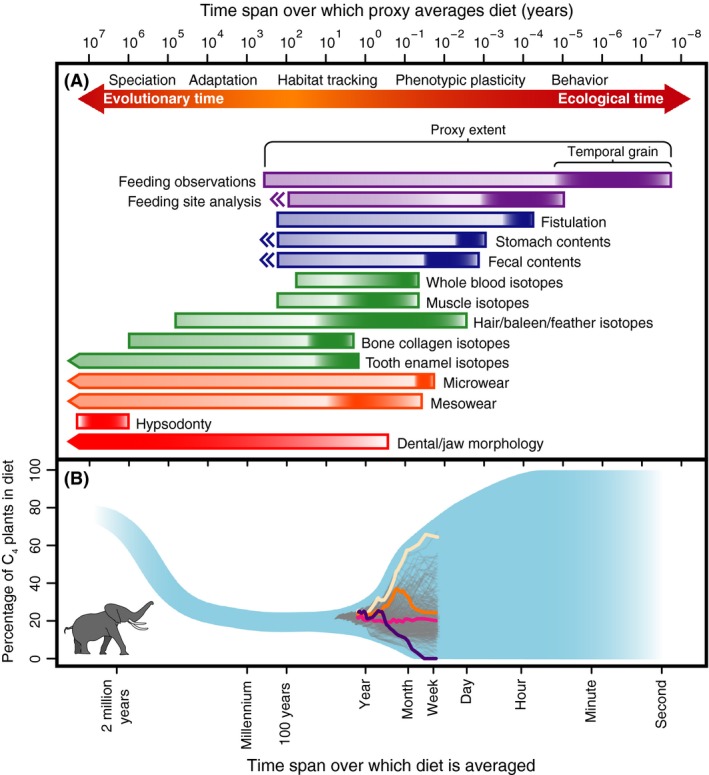
The different temporal scales over which proxies record diet. (A) A proxy's resolution is given by its temporal grain. Proxy extent is the range of time over which a proxy can be used. Proxies marked by a pointed end have ranges that extend past the graph limits. Double‐pointed ends indicate that proxies can be used in exceptional fossil cases. (B) The perceived diet of the African elephant (*Loxodonta africana*) depends on the time span over which it is measured. Blue‐shaded area represents dietary limits of elephants at different scales estimated from observational and fossil evidence. Gray lines show actual diets at different scales computed from a 6‐year isotopic record (Cerling et al. 2009). Four lines are highlighted in color to show how perceived diet changes as it is averaged for longer periods of time. Both graphs share the same logged *x*‐axis given in years above and common calendar units below.

Diet is a fundamentally important biological trait that widely influences physiology and morphology (Price and Hopkins 2015). Diet reconstructions are crucial for properly managing species, constructing food webs, studying niche theory, examining evolutionary changes in function, and inferring ancient climates and habitats (Feranec 2004; Dalerum and Angerbjörn 2005; Pineda‐Munoz and Alroy 2014). To infer natural diets, both neontologists and paleontologists have developed a series of proxies that depend on behavior, dental wear, stable isotopes, gut contents, and skeletal morphometrics (Fig. 1A). However, agreement among proxies and test diets is often poor (Kessler et al. 1981; McInnis et al. 1983; Schubert et al. 2006; Shrestha and Wegge 2006). Three proxies may show strong support for three different dietary reconstructions, confounding analysis of both fossil (Mendoza et al. 2002; Schubert et al. 2006; Figueirido et al. 2010) and extant species (Shrestha and Wegge 2006; Gogarten and Grine 2013).

This is partly because the operational meaning of “diet” is rarely explicitly defined (Hyslop 1980; Gagnon and Chew 2000). Is food ranked by the volume or mass consumed, its caloric value, or the feeding time necessary to manipulate it (Hyslop 1980; Cumberland et al. 2001)? Each proxy also records a slightly different aspect of diet. Feeding observations reveal what food enters an animal's mouth, stable isotopes record those nutrients that actually contribute to growing tissues, and fecal analysis technically only measures food that passes through the gastrointestinal tract with minimal digestion (Shrestha and Wegge 2006; West et al. 2006).

Most importantly, different dietary proxies record diet across a large range of temporal scales (Fig. 1A) (Fortelius and Solounias 2000; Dalerum and Angerbjörn 2005; Kaiser et al. 2013; Münzel et al. 2014). Stomach contents might average together up to a week's worth of meals (Kararli 1995), whereas tissues like hair record an isotopic signature of diet for as long as they are growing and could represent years or even decades of an animal's diet (West et al. 2006). Because diet can change significantly over ontogenetic (Kurle and Worthy 2002), ecological (Hobson et al. 1999; Munro et al. 2006), and evolutionary timescales (Rivals and Semprebon 2011; Rivals et al. 2012; Cerling et al. 2015), diet reconstructions can also change depending on the temporal grain and extent of measurement (Dalerum and Angerbjörn 2005; Martínez del Rio et al. 2009). Although the different temporal scales of proxies and the variability of diet are widely recognized, failing to explicitly consider them can lead to perceived incongruences in diet and questionable inferences about any related concepts such as paleohabitat reconstructions or foraging theory (Feranec 2004; Dalerum and Angerbjörn 2005; Schubert et al. 2006; Reynolds‐Hogland and Mitchell 2007). Here, we discuss the temporal scales of popular proxies and provide recommendations for their use. Adopting a cross‐scale framework that incorporates multiple proxies and stresses an explicit alignment of dietary data with the scale of one's evolutionary or ecological question allows us to roughly infer diet through almost 15 orders of temporal magnitude from seconds of an organism's life to millions of years of a lineage's evolutionary history (Fig. 1A).

## The Temporal Scale of Proxies

### Different proxies record diet over vastly different temporal scales

These scales are defined by two components: grain and extent. Temporal grain is the resolution at which a proxy can detect changes in diet (Fig. 1A). Rapid changes in diet like an animal moving between productive patches or altering foraging activity along a diel cycle can only be detected by very fine‐grained proxies such as field observations or feeding site analysis that are measuring diet over minutes to hours (Shrestha and Wegge 2006). Stable isotopes in bone collagen, however, are incorporated very slowly and could take years or decades to become completely equilibrated with a new diet (Stenhouse and Baxter 1979; Ambrose 1993; Cox and Sealy 1997). For most bones, these isotopes probably record diet at a grain not much finer than the life span of an organism (Ambrose 1993).

Temporal extent can be thought of in two ways: proxy extent and organismal extent. Proxy extent is the time span over which a proxy could be used to record diet (Fig. 1A). The stable isotopes found in tooth enamel, which are thought to hold dietary information on the order of 100 million years without significant diagenetic alteration (Zazzo et al. 2002; Koch 2007), have an incredibly long proxy extent and are useful for inferring diets from Deep Time (Kimura et al. 2013) up to the present day (Cerling et al. 2008). But fistulation, a proxy commonly used by range managers to infer the diet of domestic animals, has a very short proxy extent on the order of a century. Fistulation requires sampling ports to be surgically implanted into the gastrointestinal tracks of live animals so data extend only to the mid‐1800s when the technique was first developed and reported in the literature (Dyne and Torell 1964). Some proxies, like stomach (Kriwet et al. 2008) and fecal content analysis (Hansen 1978), can be highly informative in exceptional fossil cases but generally, they have short proxy extents of centuries or less, limited by the survival of written natural history observations. The lengths of temporal grain and proxy extent are usually correlated (Fig. 1A), but outliers like dental microwear can record weekly changes in diet over an extent of millions of years (Teaford and Oyen 1989). If masticatory dynamics are shown to be homologous among extant taxa used to calibrate discriminate functions (Mihlbachler et al. 2016), the proxy extent of dental wear features is the age of the node uniting all calibration taxa that bracket the study species in a phylogenetic tree.

Organismal extent is the time period within the life span of an organism that a proxy usefully records dietary information. Teeth will only record an isotopic signal during the few months that they are forming before becoming metabolically inert, and thus, they likely represent a juvenile diet in organisms with limited tooth replacement like mammals (Hobson and Sease 1998; Hoppe et al. 2004). Microwear patterns on teeth, however, are quickly and continuously overwritten and like gut contents require lethally invasive sampling to measure (but see Barnes and Longhurst 1960; Hyslop 1980; Teaford and Oyen 1989; Kronfeld and Dayan 1998). These proxies will only record diet during the last few days or weeks that an animal is alive (Teaford and Oyen 1989; Münzel et al. 2014). This is not a concern for extant species as stomachs can be obtained from wild caught animals throughout the year and their contents can be averaged together to generate monthly to decadal records of diet. But the Last Supper Effect in fossil microwear and stomach contents has the potential to create a taphonomic megabias in dietary reconstructions (Grine 1986). If organisms are more likely to die and enter the potential fossil record when they are consuming an atypical diet due to starvation or because they choked on abnormally recalcitrant prey, these proxies will consistently record misleading dietary information (Daegling et al. 2013). Actualistic taphonomy of primates is encouraging for the use of microwear as it shows that mortality rates are not systematically higher during periods of resource stress (Gogarten and Grine 2013), but further study is needed to evaluate these potential biases.

Unlike the quickly overwritten patterns of microwear, tooth fractures (Van Valkenburgh 2008), and mesowear that visibly changes tooth cusp shapes (Fortelius and Solounias 2000) probably reflect the rigors of diet over a substantial portion of an animal's life span and may operate at temporal scales similar to bone collagen. Ever‐growing tissues such as tusks, hair, and otoliths are not remodeled throughout the animal's lifetime like bone, so they incorporate an isotopic signal of diet only with new growth and therefore can be subsampled at finer temporal grains (West et al. 2006; Koch 2007). The time period integrated is as long as that structure has been growing, sometimes the animal's entire life span (Cerling et al. 2008) and the grain is limited only by how fast the tissue grows with daily resolutions achievable (Hoppe et al. 2004; Koch 2007).

In contrast to other proxies such as scat and isotopes, whose temporal scales can be elucidated relatively straightforwardly with diet switch experiments (Jones et al. 1981; Dickman and Huang 1988), it is harder to constrain which specific temporal grains and organismal extents a certain morphological feature represents. Although fat deposits can reflect the quality and quantity of prey in a previous year (Víkingsson 1990) and alterations in the hardness and nutrition of diet can cause significant morphological changes over an organism's lifetime (Lieberman et al. 2004; O'Regan and Kitchener 2005), sometimes in periods as short as several months, major differences in masticatory morphology like degree of hypsodonty are likely the result of many thousands or millions of years of evolution (Hummel et al. 2011; Kaiser et al. 2013). Without relatively complete, well calibrated fossil sequences and phylogenies, it is difficult to determine whether a plastic morphological feature is the result of an ancient phenotypic bottleneck that occurred over a relatively short period of time or whether it represents morphology adapted over millions of years to an average diet (Gogarten and Grine 2013). Using any morphological feature as a dietary proxy requires the difficult burden of proof that the feature is a result of long‐term adaption rather than a short‐term effect (or vice versa) and that there exists a tight linkage between form and function (Lauder 1995). We should not blindly assume this linkage. Jaws and teeth have many uses (intraspecific combat, nest building, brooding young, digging, grooming, etc.) and there are likely multiple trade‐offs and constraints that preclude them from representing *the* morphological optimum for a particular diet, especially a modern diet that is only briefly measured in the field (Lauder 1995). Gross dental or jaw morphology may be appropriate proxies for studies requiring only rough groupings of diet (i.e., carnivore vs. herbivore) that examine major evolutionary trends or adaptive radiations taking place over millions of years (Anderson et al. 2011), but they are probably best used in conjunction with other proxies as the temporal upper limit in multiscale analyses.

### Proxy scale is influenced by many factors

Even for those proxies whose temporal scales could be elucidated, very few have had parameters verified by controlled diet switch experiments (Dalerum and Angerbjörn 2005; Crawford et al. 2008; Martínez del Rio and Carleton 2012). Specific estimates of temporal grain for the widely used proxy bone collagen are extremely rare (Long et al. 2005). Reviewing the literature, Thomas and Crowther (2015) could find only two studies that actually measured the half‐life of stable isotopes in bone collagen (Hobson and Clark 1992; Carleton et al. 2008). The few experimentally derived values we have range widely, suggesting that transferring specific laboratory‐derived temporal scales to broader taxonomic groups and field conditions may be untenable (Boecklen et al. 2011; Vander Zanden et al. 2015). The lack of basic research deriving temporal scales for dietary proxies has been lamented for decades with little effect (Kaufman et al. 2008; Boecklen et al. 2011; Thomas and Crowther 2015; Vander Zanden et al. 2015).

We have provided broad estimates for proxies here (Fig. 1A) based on the best data in the literature, but the exact temporal scales that proxies record diet over can be influenced by many factors including taxonomy, physiology, mass, ontogenetic stage, sample age, tissue sampled, nutritional status, temperature, season, and the nutritional and physical properties of food (Kaufman et al. 2008; Boecklen et al. 2011; Ben‐David and Flaherty 2012; Martínez del Rio and Carleton 2012; Mihlbachler et al. 2016). For example, without difficult to construct correction factors (Dickman and Huang 1988), food that takes longer to digest will be overrepresented in diet reconstructions as it will dominate handling time observations and remain identifiable longer in gut and fecal analysis (McInnis et al. 1983; Shrestha and Wegge 2006; Pineda‐Munoz and Alroy 2014). Even consuming the same food, young, growing individuals will incorporate nitrogen isotopes faster than older individuals that are recycling nitrogen from body stores (Ben‐David and Flaherty 2012). And regardless of nonhomologous dental facets between taxa, microwear might still have a longer temporal grain for ruminant artiodactyls compared to other ungulates because wear features are formed by chewing cud, which is predigested in the rumen, instead of freshly obtained forage (Mihlbachler et al. 2016).

Mass alone could cause major differences in isotopic incorporation rates (Thomas and Crowther 2015; Vander Zanden et al. 2015), a pattern, that is, partially (Tieszen et al. 1983) but not completely (Boecklen et al. 2011) explained by metabolic scaling theory. All things being equal, a nearly 3500 kg elephant (*Loxodonta africana*) would take about 2.7 years to fully reflect a dietary shift in the stable carbon isotopic composition of its muscle while even something as light as a 38 g striped mouse (*Lemniscomys striatus*) would take around 107 days (Thomas and Crowther 2015). Larger organisms, in effect, record diet more slowly than smaller organisms and subsequently sample the landscape at a longer temporal grain. If the integration time of a tissue is long compared to temporal variation in diet, conditions that are almost guaranteed for most larger animals, the measured isotopic composition of that tissue will always be out of equilibrium with the actual diet, violating one of the major assumptions required for isotopic dietary reconstruction (Carleton et al. 2008; Ben‐David and Flaherty 2012; Phillips et al. 2014). The effect holds even for dietary specialists as identical prey or forage items can exhibit variable nutrient and isotopic compositions over time (Ben‐David and Flaherty 2012).

## Different Temporal Scales Lead to Perceived Incongruences in Diet Reconstructions

### One proxy may have different scales for different taxa

**Figure 2:**
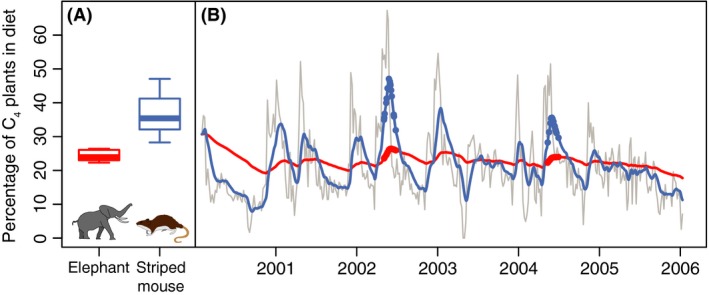
Hypothetical example illustrating the effects of temporal averaging on diet and how it can cause the same diet to appear different in two species. (B) The “true” percentage of C_4_ plants consumed taken from a 6‐year isotopic record (Cerling et al. 2009) of African elephants (*Loxodonta africana*) is shown by the gray line. If elephants and striped mice (*Lemniscomys striatus*) each consumed the same diet shown by the gray line, different mass‐related turnover times would cause stable isotopes in their muscle tissue to produce the different “measured” diets shown by the red and blue lines, respectively. If an ecologist sampled elephant and mouse muscle tissue on 15 random days in the late spring in two separate years (circles), she would reconstruct elephants and mice as having very different dietary distributions, (A) even though they were consuming the exact same percentages of C_4_ plants at the same time.

Elephants are a threatened species and major ecosystem engineers (Johnson et al. 1999), so knowing their actual diet is an important part of conservation efforts as well as paleohabitat reconstructions (Feranec 2004; Schubert et al. 2006; Cerling et al. 2015). Imagine an ecologist suspects that foraging by elephants impacts the diet of striped mice. She could sample the ^13^C values of elephant and mice muscle tissue in Samburu National Reserve in Kenya and find that each species consumes a different proportion of C_4_ (tropical grasses) vs. C_3_ (trees, shrubs, and forbs) vegetation (Fig. 2A). Depending on the diet of striped mice in a control enclosure, she might conclude that elephants have competitively excluded striped mice from the browse region of dietary space or that the mice consumed low quantities of C_3_ vegetation regardless of elephants. She would not conclude that they currently have identical diets. But the isotopes measured in the muscle tissue of striped mice and elephants are recording diet over very different timescales. Both species could consume identical meals but display nonoverlapping diets as measured by the isotopic composition of their tissues.

To show this, we took a 6‐year isotopic record of elephant diet made from serially sampled tail hair (Cerling et al. 2009) and linearly interpolated the near weekly resolution into daily values to represent the “true,” identical diet of elephants and striped mice (Fig. 2B). Two negative percentages in the original data were set to zero as they are impossible and an artifact of the mixing model used by Cerling et al. (2009). Applying the ^13^C‐mass‐specific half‐lives for mammals given by equation (6) in Thomas and Crowther (2015) for 3940 kg elephants and 38.1 g striped mice (Tóth et al. 2014) and assuming simple first‐order kinematics produces very different “measured” diets that would be obtained from muscle tissue (Fig. 2B). If the ecologist sampled tissues from elephants and mice at the same time on 15 random days during the months of May and June during her 2002 and 2004 field seasons (Fig. 2B) (a reasonable scenario given poaching, trapping, and natural deaths), she would produce the distributions in measured diets shown in Figure 2A, diets which do not match each other or the true intake of C_4_ plants during this interval.

**Figure 3:**
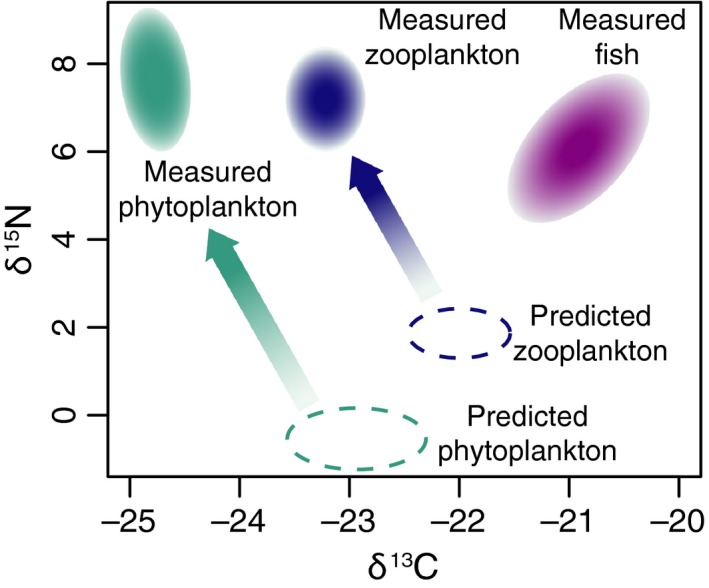
Diagrammatic example based off of O'Reilly et al. (2002) showing a simple food chain in Lake Tanganyika, East Africa that is flipped upside down with the measured isotopic values of phytoplankton and zooplankton making them appear more trophically enriched than their predators, fish. The anomaly was explained by lower trophic levels recording a recent upwelling of nitrate that had not yet been integrated into fish muscle, a tissue that records dietary changes much more slowly than the full body isotopes of phytoplankton and zooplankton.

This may seem like a contrived example, but O'Reilly et al. (2002) found a similar pattern when they examined the muscle and whole‐body isotopes of organisms in Lake Tanganyika, East Africa, and saw a simple pelagic food chain flipped upside down with phytoplankton and zooplankton more trophically enriched than the fish that consumed them (Fig. 3). The anomaly was explained by lower trophic levels recording a recent upwelling of nitrate that had not yet been integrated into fish muscle. Phytoplankton and zooplankton reflect their environment at a finer temporal scale than fish do. Even when using identical proxies, different time averaging among species requires consideration (O'Reilly et al. 2002).

Although the mechanisms for time averaging are different for each proxy, they will all lead to similar effects on measured diets. Longer time averaging will inevitably cause the range and variance of diets to decrease. In proxies like whole‐body isotopes, this would manifest as larger species appearing to have a more restricted diet than populations of smaller organisms, which will show greater intraspecific variation. As illustrated in the example of the elephant and striped mouse above, different time averaging can make species with identical diets appear to eat different things. It can also do the converse, causing dissimilar diets to appear similar. Temporal weighting of the average will lead to diets that are out of phase with any cyclical pattern and peaks and valleys in resource consumption will always be smoothed to some degree, leading to under‐ or overprediction of resource use or availability. Any step change in diet would appear more gradual, or even nonexistent, if the proxy averages so much time that the change is stretched out longer than the sampling period. Longer temporal averaging of diet can also change the inherent spatial scale of analysis with food potentially coming from multiple forage patches or even endpoints of a transcontinental migration (Bauchinger and McWilliams 2009). None of these biases would be apparent if diet were constant, but the high variability of diet from short to long timescales means that proxies, at any grain, could be tracking the moving target of a diet at disequilibrium (Carleton et al. 2008; Ben‐David and Flaherty 2012). Dietary proxies will never fully reflect the instantaneous consumption of an organism.

### Different proxies may predict different diets for the same organism

Even when using proxies to measure only one species, the different temporal averaging of proxies can lead to large and seemingly inexplicable incongruences in diet reconstructions (Gogarten and Grine 2013). Continuing our example, elephants possess large teeth with grooved ridges that are continually replaced in a conveyor belt fashion (Laursen and Bekoff 1978), features thought to be adaptations to a diet of massive quantities of grass (Cerling et al. 1999). However, isotopic work on modern elephants shows that they are browsers, consuming little graze (Cerling et al. 1999). Descriptions of elephant diet based on stomach contents and field observations further complicate matters as they vary widely, even when considering elephants in the same region (Cerling et al. 1999). Using the same tail hair isotopic record of Cerling et al. (2009), we started at the last date measured (11 January 2006) and computed successive cumulative averages representing diet for ~ 1 week, 2 weeks, 3 weeks, and so on until the dietary signal was averaged for the full time span of the study, almost 6 years (Fig. 1B). This reveals how much diet can change based only on how long it is measured for. But this line of successively larger cumulative averages is heavily influenced by starting conditions and would change depending on whether we began measuring an elephant's diet during the rainy season when it was consuming more grass or during the dry season when it was consuming more browse. We therefore repeated this process for each measured time point in Cerling et al. (2009) producing 367 lines of decreasing length that show how an individual's diet could change if it were averaged over longer and longer time spans (Fig. 1B). Assuming simple unweighted averaging, stable isotopes from a 5 mm clipping of tail hair would reveal that for the week around 18 May 2002 an elephant ate 67% C_4_ plants, making it a grass‐dominated mixed feeder in most classifications (Cerling et al. 2009). But if we sampled the stable isotopes in the collagen of a 5‐mm‐radius transverse slice from that same elephant's tusk, we would find that it ate only 24% C_4_, making it a browser (Codron et al. 2012). This large incongruence exists only because we are averaging diet for a single week with one proxy and for 70 weeks with the other; both record the same diet.

The best dataset available (Cerling et al. 2009) only covers a small portion of the range of scales over which we might investigate diet. But it would be trivial to find an elephant eating 100% browse or graze during any given minute (Cerling et al. 1999), so at shorter time spans, we would expect estimates to cover the full range of possibilities between grazer and browser (Fig. 1B). Isotopes from fossils (Cerling et al. 1999, 2015) show that elephants ate a much higher percentage of C_4_ plants in the past than they do now, so we would also expect an upswing in C_4_ plant consumption if diets were averaged for millions of years (Fig. 1B). This is why the morphology of modern elephant teeth, a very coarse‐grained proxy, suggests a grazing diet, whereas isotopes of those same teeth suggest browsing affinities, and why observations of elephants in the field can show them eating both browse and graze. Each proxy reflects the true diet measured over a specific time span.

## No One Scale or Proxy is Correct

### Different proxies answer different questions

The fact that the different temporal scales of proxies require consideration does not mean that they are a problem. No one scale is more correct than any other. The long herbivorous evolutionary history of artiodactyls does not refute film from remote cameras showing that they can selectively depredate bird nests in a matter of seconds (Bazely 1989; Pietz and Granfors 2000). But any such rapid dietary variation would be nothing but noise when investigating how historic whaling and the Pleistocene megafaunal extinction changed terrestrial resource use of condors over thousands of years (Chamberlain et al. 2005). The utility of different proxies depends on the nature of the research question and the temporal scale examined (Kronfeld and Dayan 1998).

### Diet should be viewed at multiple scales

The richest understanding of diet, however, comes not from one particular scale but from comparing diet across several scales (Tieszen et al. 1983; Dalerum and Angerbjörn 2005; Schubert et al. 2006; Bauchinger and McWilliams 2009; Martínez del Rio et al. 2009). Using a cross‐scale framework, researchers can compare diet at different times and over different time spans, measuring not what an organism's diet *is* but rather how it changed. This is a powerful framework that has been effectively used to investigate a wide range of problems from the syntopy of congeneric birds through seasonal differentiation of marine resource use (Martínez del Rio et al. 2009) to the origin of human remains and whether they were newly captured slaves from different villages at their time of death (Cox and Sealy 1997).

Cross‐scale estimates of diet can be generated by repeated sampling of a proxy over time, comparing proxies with different temporal scales, and serially subsampling a tissue that integrates diet over time (Dalerum and Angerbjörn 2005). Sampling one proxy repeatedly over time is the most common form of cross‐scale dietary analysis (Dalerum and Angerbjörn 2005) and can measure seasonal and yearly variation (Andelt et al. 1987) or major trends over millions of years (Kimura et al. 2013) depending on whether the proxy has a short temporal extent like fecal contents or a long extent like dental isotopes.

Comparing differently scaled proxies in the same species is the least common method of constructing cross‐scale descriptions among modern ecologists (Dalerum and Angerbjörn 2005), but it should be used more often, especially in conservation studies, because a wide range of dietary timescales can be sampled nonlethally during just one encounter (Martínez del Rio et al. 2009). Differences in proxy values can be used to back calculate the time elapsed since a diet shift (Bauchinger and McWilliams 2009), but this “isotopic‐clock” methodology requires the assumption of a step change between equilibrium diets, making it impractical for most field situations (Phillips and Eldridge 2005) except possibly major dietary shifts like weaning (Richards et al. 2002). Multiproxy analysis is already common in paleontological studies and is increasingly used in a temporally explicit framework to understand diet from ecological to evolutionary timescales (Rivals et al. 2007; Semprebon and Rivals 2010; Louys et al. 2012; Tütken et al. 2013).

Serially sampling tissues that have progressive growth such as hair, baleen, tusks, otoliths, and some feathers has the greatest potential to create long‐term, high‐resolution dietary records (Cerling et al. 2009) as some tissues could record decades of diet with almost weekly resolution (Schell 2000; Dalerum and Angerbjörn 2005). If isotopic baseline issues are accounted for (Long et al. 2005; Casey and Post 2011), high‐resolution dietary records tracking anthropogenic climate change through decades or even centuries could be created by linking together tusk or baleen data from multiple museum specimens (Schell 2000). Otoliths, most commonly used as a proxy in teleost fishes, could potentially provide high‐resolution dietary data going back much further, at least hundreds of millions of years (Patterson 1999; Koch 2007), but their isotopic values can be heavily and unpredictably influenced by dissolved inorganic carbon and likely reflect the composition of water more than a pure dietary signal (Walther and Thorrold 2006; McMahon et al. 2011). Some techniques have been proposed to minimize potential nondiet biases (McMahon et al. 2011; von Biela et al. 2015), but more basic research is needed before otoliths can be confidently used in extant or fossil fishes as a dietary proxy (Elsdon et al. 2010).

## Toward an Explicit Cross‐Scale Dietary Framework

### There are numerous barriers to a cross‐scale approach

Unfortunately, as a field, we do not have enough data to adopt rigorous multiscale approaches in all analyses. Although some taxa like elephants are well studied with multiple proxies (Cerling et al. 1999), many species lack any kind of dietary data (Penone et al. 2014). Despite a broad acceptance of the temporal variability of diet (Dalerum and Angerbjörn 2005), most models implicitly assume diet stationarity, assigning a single qualitative variable like “browser” or “omnivore” whether examining trophic guilds over millions of years or a small forest plot through one day (Wilman et al. 2014). Although criticized for having arbitrary boundaries (Feranec 2004; Pineda‐Munoz and Alroy 2014), these rough diet categorizations are comparable across taxonomic groups and studies and are simpler to incorporate into complex statistical models (Price et al. 2012). They are also often the only dietary information available.

Collecting data from proxies like stomach contents is difficult and prohibitively expensive (Kessler et al. 1981; McInnis et al. 1983). Even compiling data from the literature is complicated as experts often do not express diet quantitatively or use idiosyncratic classification schemes that may have suited their original studies but are now difficult to compare, for example, percent seeds vs. leaves and percent graminoids vs. forbs (Gagnon and Chew 2000; Wilman et al. 2014). Most studies considering more than a few species must rely on previously compiled datasets that synthesize information from many proxies, scales, and sources into rough measures of diet (Gagnon and Chew 2000; Cerling et al. 2003; Wilman et al. 2014). Even then, many species lack dietary data. Price et al. (2012) were only able to find primary reports of diet obtained from direct observation or stomach and fecal contents for 30% of extant mammals, one of the best studied major taxonomic groups. PanTHERIA (Jones et al. 2009), a larger effort using additional proxies could only assemble data for 40% of included species. Even using more numerous qualitative sources of data, EltonTraits 1.0 (Wilman et al. 2014) found diets for at most, 81% of extant mammal species. Only PanTHERIA (Jones et al. 2009) explicitly lists the temporal scale of measurement and only for some species. This makes it difficult to account for temporal scaling in downstream analyses when using these dietary compilations, an omission that may introduce severe errors (Dalerum and Angerbjörn 2005; Losos 2008).

### Different subfields favor different proxies

Most proxies are also used out of operational necessity not for a preferred temporal scale of investigation (Reynolds‐Hogland and Mitchell 2007). While feeding observations are commonly employed to study extant animals, the proxy is impossible to apply to fossil species excepting what can be gleaned from prehistoric cave art (Guthrie 2000). Proxies such as feeding site analysis (Rawn‐Schatzinger 1992), scat (Hansen 1978), and stomach contents (Kriwet et al. 2008) can be used with extinct species, but methods involving the morphological or isotopic examination of dentition have a much larger potential sample size relying only on the robust fossil record of isolated teeth (Koch 2007; Kimura et al. 2013). While all the dietary proxies discussed here are feasible to use with extant animals, certain proxies require destructive sampling and would be unsuitable for use on elusive or endangered species (Pineda‐Munoz and Alroy 2014). This is why most conservation biologists study diet through noninvasive methods such as feeding observations or sampling dung and shed hair (Martínez del Rio et al. 2009). No one could justify killing an endangered species to take isotopic samples of bone collagen, but for a fossil species known only by fragmentary postcrania, bone collagen is the only proxy possible.

**Figure 4:**
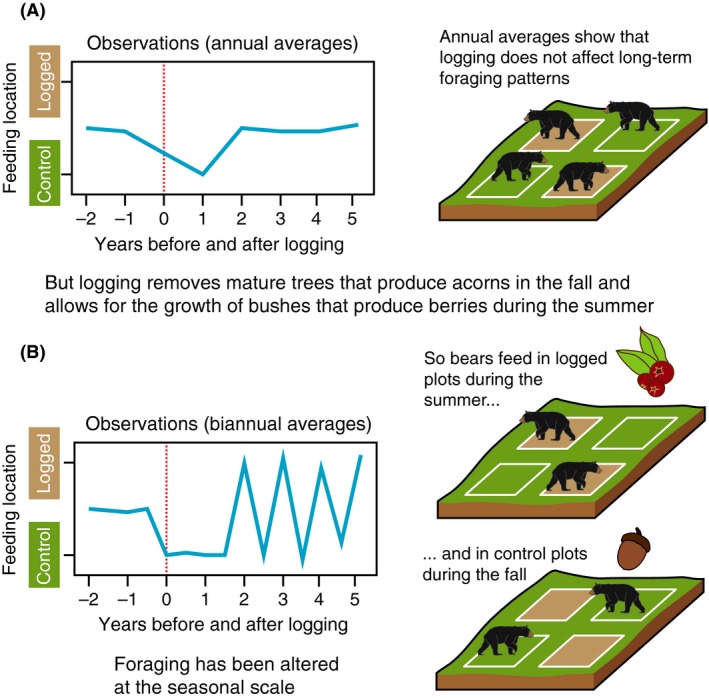
Diagrammatic example based off of Reynolds‐Hogland and Mitchell (2007) showing how changes in the temporal grain of an investigation can completely alter the conclusions of a study. (A) Logging seems to have no long‐term effect on American black bear (*Ursus americanus*) foraging patterns but a better understanding of the study system shows that yearly averages are not the correct scale to capture the biological and behavioral changes that logging brings. (B) Seasonal or biannual averages reveal that logging greatly alters bears' foraging patterns.

These different operational limitations force researchers to favor certain proxies over others creating a nonrandom bias in which temporal scales of diet are considered by each disciplinary subfield (Fig. 1A). Using mostly coarse‐grained proxies, paleontologists can underestimate dietary variability at shorter timescales, leading to erroneous paleohabitat reconstructions based only on a portion of a species' diet. Neontologists, using mostly proxies with shorter extents, underestimate the fundamental niche and dietary variability at longer timescales, producing models that may not accurately predict ranges under climate change or anthropogenic threats. If these biases remain unacknowledged, they hamper our understanding of general ecological and evolutionary patterns by reducing meaningful communication between subfields. Even if we use a proxy because it is the only method available, it is crucial to understand the scale at which that proxy records diet so that we can ensure that the scale of our question does not exceed that of our data (Fig. 4) (Behrensmeyer 2006; Reynolds‐Hogland and Mitchell 2007; Crawford et al. 2008).

## Recommendations for Research Involving Dietary Data

### Explicitly state the temporal scale of your question

This will dictate the necessary proxy extent, organismal extent, and grain of your dietary proxies (Reynolds‐Hogland and Mitchell 2007; Bennington et al. 2009; Thomas and Crowther 2015). Are you concerned with seasonal variability in diet? Your proxy has to have a finer grain than the seasons you are hoping to measure (Fig. 4). How many years do you need to measure to generalize the periodicity of seasons? This is the duration of your study system. Are you interested in seasonal variability in the deep past or in the present day? Each suggests a different proxy. Be aware that just because a proxy has a fine grain, it does not mean that sampling is at an equally fine grain (Bennington et al. 2009). Stomach contents, representing a week of diet, might only be collected twice a year and even low amounts of time averaging in a fossil assemblage will negate the weekly resolution of dental microwear. Seasonal variation will still be measured; it will just be difficult to attribute to seasonal forcing.

### Explicitly state the scale of your proxies

Whether your proxies are chosen out of operational necessity or because they cover the optimal temporal scale to answer your question, their scales need to be explicit (Bennington et al. 2009). Recognize that the exact temporal scale that a proxy operates over is likely unknown or maybe even unknowable (Reynard and Tuross 2015; Thomas and Crowther 2015). Still, an order of magnitude estimate of proxy grain and organismal extent should provide more clarity than a scale blind approach. Be cautious using values from the literature, which may not be generalizable to other taxa or environments (Boecklen et al. 2011; Vander Zanden et al. 2015). Back up your estimates of proxy scale with sound justifications and point out factors that may lead to other values. Are there morphological or physiological differences between taxa that could affect the scale of a proxy (Long et al. 2005; Ben‐David and Flaherty 2012; Mihlbachler et al. 2016)? Work these estimates into a sensitivity analysis and provide at least qualitative error estimates of how changes in scale could affect downstream analyses. For example, are your isotopic results robust to a range of estimates for body mass and internal temperatures (Thomas and Crowther 2015)? Scale effects could be larger than expected and completely change the conclusions and significance of your results (Long et al. 2005; Reynolds‐Hogland and Mitchell 2007). Don't ignore organismal extent. If you are using dental isotopes or microwear of a long‐lived organism, you may be recording atypical diets at the extreme endpoints of its life span, not the diet during the majority its ecological agency (Grine 1986; Hobson and Sease 1998; Münzel et al. 2014). Even a proxy like bone collagen that measures diet over the life span of an organism is likely heavily weighted towards diet early in life and during periods of growth (Cox and Sealy 1997; Hedges et al. 2007).

### Favor a multiscale approach that utilizes a range of proxies

This balances the methodological strengths and weaknesses of individual proxies allowing, for example, the separation of C_3_ browsers and C_3_ grazers, which cannot be detected by isotopic means alone (Schubert 2007). But most importantly, it allows for a richer view of diet (Tieszen et al. 1983; Dalerum and Angerbjörn 2005). Proxies likely will not agree. This perceived incongruence is not a problem but an opportunity to understand how diet has changed over temporal scales (Bennington et al. 2009). Treating elephants as yearly browsers, evolved from grazing ancestors, which increase their consumption of C_4_ grasses during the rainy season is a much more holistic and heuristic view for both paleohabitat reconstruction and game management than just arguing over whether browse or graze is the “true” diet.

### Recognize when you are implicitly using a multiscale approach

Using an identical proxy for all species in your analysis does not necessarily mean that results are directly comparable between taxa (Figs. 2, 3). One proxy, like dental isotopes, may actually operate over a range of temporal grains and organismal extents depending on taxonomy or environmental factors (Ben‐David and Flaherty 2012). Even recording the same diet, teeth that average diet over longer periods will show less measured variation than faster growing teeth. Will your results be affected if one species' teeth have finished growing before weaning while another's have not? Isotopic samples taken from historical specimens and long‐lived organisms (Lee et al. 2005) also need to be corrected for changing atmospheric concentrations of ^13^C and ^15^N and lagged incorporation of those concentrations that depends on trophic level, life span, and primary producer identity (Long et al. 2005; Casey and Post 2011). Carnivores only incorporate atmospheric isotopes that have first been fixed by plants and then eaten by and incorporated into herbivores so they could be recording atmosphere from years before other species in a dataset; detritivores would reflect atmosphere even further in the past (Long et al. 2005). Make sure the results you are plotting can be directly compared between taxa (Figs. 2, 3).

Also be mindful of the related issue of “scale jumping,” inadvertently comparing data sources from very different scales (Behrensmeyer 2006; Behrensmeyer and Reed 2013). You might use the popular WorldClim database (Hijmans et al. 2005) to test a relationship between climate and the contents of a large number of frog stomachs collected in 1905. But WorldClim bins climate data between 1950 and 2000. How much would climate change and averaging temperatures over 50 years affect your relationship? The largest and most common scale jump probably occurs while developing dietary proxies based on gross morphology. Morphological features such as jaw length and depth, which could take millions of years to evolve are compared to categorical diets such as “browser” or “grazer” which have been generated from possibly only a year of stomach content or observational data (Gagnon and Chew 2000). Even using phylogenetic discriminate analysis to construct models (Motani and Schmitz 2011), there must be extreme plasticity and selection for similar adaptive morphological peaks or a relatively constant diet over very long time spans for these relationships to hold. Taxonomic uniformitarianism might work for some groups with clearly specialized morphologies and diets, but it has been shown increasingly untenable as a null model for many others (Emslie and Patterson 2007; Faith 2011; Cerling et al. 2015). Far better would be to compare long studies of microwear or isotopes, whose extents may exceed the grain of morphology, to the features you wish to study. Showing that a morphological proxy seems to hold over an extremely broad group of taxa also makes diet reconstructions more robust as it suggests a tight physical optimum with temporal scales that can be constrained, at least roughly, through phylogeny.

### Understand the entire proxy system

Before we can understand how numerous behavioral, physiological, climatological, and taphonomic inputs may affect the temporal scales of proxies in complex field situations, we need more basic research in controlled environments (Kaufman et al. 2008; Boecklen et al. 2011; Mihlbachler et al. 2016; Price 2015; Thomas and Crowther 2015; Vander Zanden et al. 2015). Diet switch experiments and investigations of historical museum specimens would provide us with a much better understanding of the range of intraspecific dietary variability that can be found in cross‐scale analyses. A single museum specimen could provide observational data in the form of field notes, stomach contents, dental microwear and mesowear, tooth isotopes, bone collagen isotopes, muscle isotopes, serially sectioned hair isotopes, and tooth morphology all linked to a spatially and temporally explicit location. However, isotopic values would have to be corrected for the known effects of different preservatives such as ethanol and formalin (Bugoni et al. 2008; González Bergonzoni et al. 2015).

Of course, we will only be able to experimentally examine proxies in a limited number of extant taxa and conditions. We cannot do a diet switch experiment with an extinct giant ground sloth. But that does not mean that we can blindly use popular proxies such as dental microwear, muzzle width, and hypsodonty index on extinct taxa and assume that results are comparable to those from the modern artiodactyls that these proxies were calibrated on. Ground sloths, like all xenarthrans, lack enamel on their teeth, and they have grasping manus instead of hooves. These morphological features, as well as environmental, physiological, and behavioral traits that could impact the dietary signal and timescale that proxies record, need to be carefully investigated before we can interpret proxy data accurately (Bargo et al. 2006a,b; Haupt et al. 2013). We have to rely on first principles, phylogenetic bracketing, rare natural experiments, and cautious interpretation of data, recognizing that there is no such thing as a taxon (or environment/trophic level/physiology/etc.)‐free proxy (Mihlbachler et al. 2016; Reynard and Tuross 2015).

### Consider the effects of scale at every step of research

As mentioned above, any experimental design, whether in the laboratory or the field, should account for, and align, temporal, spatial, and ecological scales (Reynolds‐Hogland and Mitchell 2007). Decisions of scale from data collection through analysis and conclusions should be explicit and justified given the parameters of your questions, data, and the ecology of organisms under study. Changing the extent or grain of a dietary measurements in an ecological study can completely change its results, the generality of its conclusions, and the formulation of any downstream management considerations (Fig. 4) (Reynolds‐Hogland and Mitchell 2007). Often, we do not know at what scale an ecological phenomena operates over a priori or we are handed the scales we must use by taphonomic or logistical realities (Reynolds‐Hogland and Mitchell 2007; Bennington et al. 2009). Still, carefully designed and analyzed experiments using sound biological reasoning that considers the whole proxy system and how multiple inputs may affect scale can account for these factors (Reynolds‐Hogland and Mitchell 2007; Ben‐David and Flaherty 2012). Considering scale throughout all aspects of research is not simple or easy, but it is necessary, and it makes our conclusions more robust even if scale constraints force them to be less exciting (Reynolds‐Hogland and Mitchell 2007).

## Conclusions

**Figure 5:**
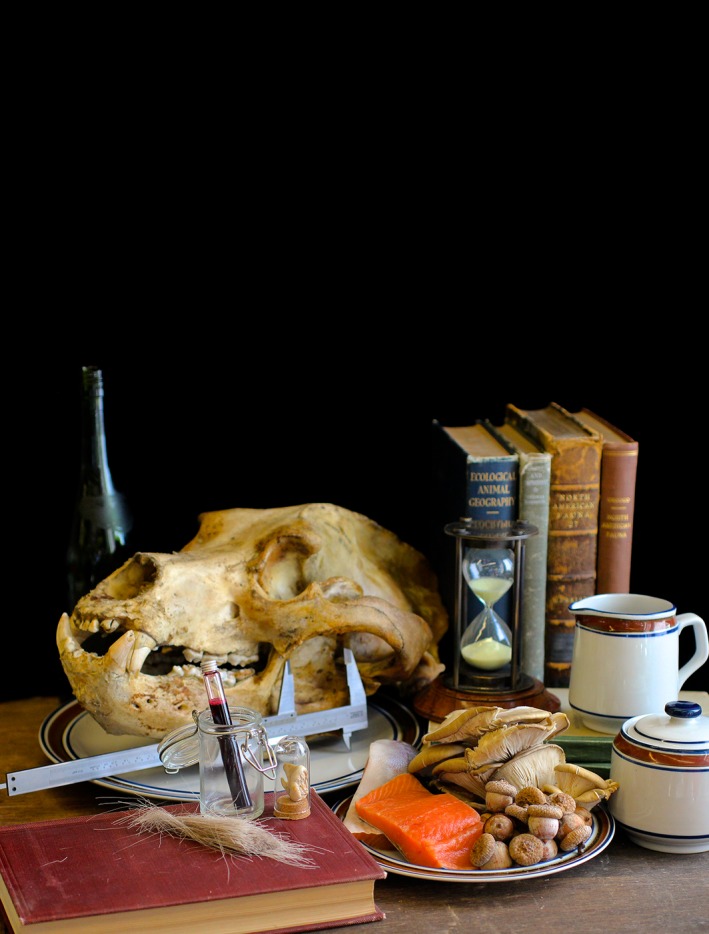
Still life of grizzly bear (*Ursus arctos*) with diet inferred from multiple dietary proxies like gross morphology and isotopes of hair, teeth, and blood. Division of Vertebrate Zoology, YPM MAM 9751. Courtesy of the Peabody Museum of Natural History, Yale University, New Haven, CT, USA.

Asking what an organism eats is a question with an inherent temporal scale (Fig. 5) (Phillips et al. 2014). Without specifying when and for how long diet is measured for, the question makes no more sense than asking what the length of the coastline of Great Britain is without specifying the length of the ruler used (Mandelbrot 1967). Just as spatial ecologists recognize that biological patterns vary across spatial scales (Belmaker and Jetz 2010), we need to change how we conceptualize traits like diet that vary across temporal scales (Wolkovich et al. 2014). It is only by carefully validating proxies and thoughtfully aligning the scale of our questions with the scale of our data that we will avoid problems inherent in the various temporal scales of diet and dietary proxies (Bennington et al. 2009; Ben‐David and Flaherty 2012). In many cases, sensitivity analysis may reveal that different temporal averaging does not significantly affect results, but as we have shown here, using a scale blind approach can lead to misunderstandings and perceived incongruences. Results from any dietary analysis need to be interpreted with caution. Rather than problems to be worked around though, we should view the different temporal scales of proxies as incredibly useful tools that bring us to a richer understanding of diet, one that stretches from an instant in an organism's life to millions of years of evolutionary history.

## Conflict of Interest

None declared.
